# Requirement of TORC1 for Late-Phase Long-Term Potentiation in the Hippocampus

**DOI:** 10.1371/journal.pone.0000016

**Published:** 2006-12-20

**Authors:** Yang Zhou, Hao Wu, Shuai Li, Qian Chen, Xue-Wen Cheng, Jing Zheng, Hiroshi Takemori, Zhi-Qi Xiong

**Affiliations:** 1 Institute of Neuroscience and Key Laboratory of Neurobiology, Shanghai Institutes for Biological Sciences, Chinese Academy of Sciences Shanghai, China; 2 Graduate School of the Chinese Academy of Sciences Shanghai, China; 3 Laboratory of Cell Signal and Metabolism, National Institute of Biomedical Innovation Ibaraki, Osaka, Japan; Emory University, United States of America

## Abstract

Late-phase long-term potentiation (L-LTP) and long-term memory depend on the transcription of mRNA of CRE-driven genes and synthesis of proteins. However, how synaptic signals propagate to the nucleus is unclear. Here we report that the CREB coactivator TORC1 (transducer of regulated CREB activity 1) undergoes neuronal activity-induced translocation from the cytoplasm to the nucleus, a process required for CRE-dependent gene expression and L-LTP. Overexpressing a dominant-negative form of TORC1 or down-regulating TORC1 expression prevented activity-dependent transcription of CREB target genes in cultured hippocampal neurons, while overexpressing a wild-type form of TORC1 facilitated basal and activity-induced transcription of CREB target genes. Furthermore, overexpressing the dominant-negative form of TORC1 suppressed the maintenance of L-LTP without affecting early-phase LTP, while overexpressing the wild-type form of TORC1 facilitated the induction of L-LTP in hippocampal slices. Our results indicate that TORC1 is essential for CRE-driven gene expression and maintenance of long-term synaptic potentiation.

## Introduction

Long-term potentiation (LTP) of synaptic transmission is an attractive cellular mechanism for learning and memory [1], [2]. Like memory, LTP can be divided into two distinct phases, an early-phase LTP (E-LTP) that depends on the modification of pre-existing proteins, and a late-phase LTP (L-LTP) that requires synthesis of mRNAs and proteins [3]–[5]. The molecular mechanisms underlying the formation and consolidation of long-term memory and plasticity in both invertebrates and vertebrates has been intensively studied during the last decade [4], [6]–[10]. These studies established the pivotal role of gene transcription mediated by CREB family transcriptional factors and its coactivators in several forms of long-term plasticity and memory in a variety of species [4], [7], [8], [11]–[13]. Phosphorylation of CREB at Ser133, triggered by Ca^2+^ or cAMP signaling, leads to the recruitment of its coactivators CBP and p300 to the CRE element and promotes the transcription of downstream genes [14]–[18]. The convergence of cAMP and Ca^2+^ signals at the level of CREB Ser133 phosphorylation provides a plausible mechanism for cooperativity among diverse signals for CREB target gene transcription and synaptic plasticity. However, recent findings have challenged this model and argued for the involvement of additional CREB coactivators in mediating CRE-driven gene transcription [4], [12], [16], [18]. For example, CREB DNA binding/dimerization domain (bZIP) contributes significantly to CRE-mediated gene expression in response to membrane depolarizing signals, implicating this domain in mediating the association of CREB with a calcium-regulated coactivator [19]. Several groups reported that some extracellular stimuli capable of phosphorylating CREB on Ser-133 fail to induce CREB-dependent gene expression [12]. Furthermore, studying LTP using CRE-LacZ reporter mice revealed the discrepancy between CREB phosphorylation status and CRE-driven gene transcription in hippocampal slice preparation [4]. These findings raised the possible involvement of other coactivators working cooperatively with CREB for activity-dependent CRE-target gene transcription. Efforts to identify novel CREB coactivators led to the discovery of a conserved family of modulators called transducers of regulated CREB activity (TORCs) [20], [21].

Functional TORC genes were identified in *Drosophila*, mouse and human genomes. Nuclear translocation of TORCs enhances CREB-dependent gene transcription via interaction with the bZIP domain of CREB [22], [23], and plays a critical role in controlling fasting glucose metabolism in mice [24]. To gain insight into the potential function of TORCs in the central nervous system, we cloned the TORC isoforms from the adult rat brain and found one of the isoforms TORC1 is highly expressed in rat hippocampal neurons. We found that TORC1 undergoes neuronal activity-dependent nuclear translocation and is required for CREB-target gene transcription in neurons. Blocking the functional interaction of TORC1 with CREB prevented the transcription of CRE-driven gene and L-LTP maintenance, while overexpression of TORC1 lowered the threshold for L-LTP induction. These data indicate that TORC1 is essential for CRE-driven gene transcription and maintenance of L-LTP in the hippocampus.

## Results

### Expression of TORC1 in rat hippocampal neurons

**Figure 1:**
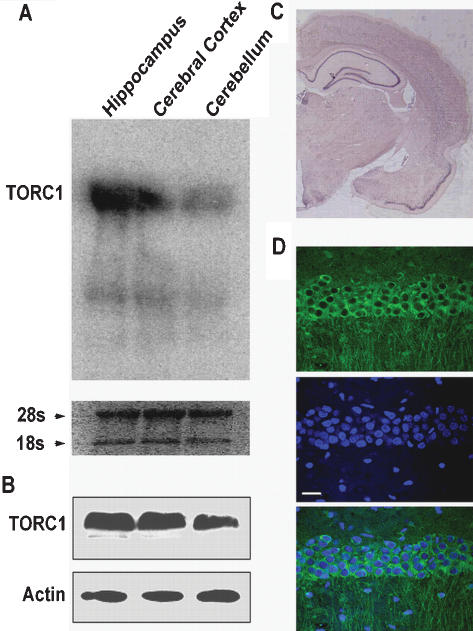
Expression pattern and subcellular distribution of TORC1 in rat hippocampal neurons. (A) Northern blotting analysis of TORC1 mRNA in the hippocampus, cerebral cortex, cerebellum of adult rat brain. 28S and 18S RNA were used as a control for RNA loading. (B) Western blotting of protein extracts from hippocampus, cerebral cortex, cerebellum of adult rat using TORC1 antibody. Equivalent protein loading was confirmed by probing the same blots with beta-actin antibody. (C) *In situ* hybridization analysis of TORC1 mRNA expression from coronal section of adult rat brain. (D) Immunohistochemical analysis of TORC1 subcellular distribution in CA1 region of rat hippocampal neurons (upper panel), Hochest 33324 was used for nuclear staining (middle panel), merged image (lower panel). Scale bar: 20 µm.

We cloned the TORC isoforms from the adult rat brain by reverse transcriptase-dependent polymerase chain reaction (RT-PCR). Both TORC1 mRNA and protein were highly expressed in the hippocampus, cerebral cortex, and cerebellum (Figure 1A and 1B), while TORC2 was mainly expressed in the cerebellum and TORC3 was undetectable in these three regions (Figure S1). Sequence analysis revealed that rat TORC1 gene (GenBank accession: 108865216) contains a 1893-base pair open reading frame that encodes 630 amino acid residues, with a molecular weight of approximately 67 kDa. Sequence comparison showed that TORC1 is highly conserved in human, mouse and rat, with 88% identity at amino acid level (Figure S2). *In situ* hybridization study of TORC1 further revealed that TORC1 mRNA was highly expressed in principal neurons of the rat hippocampus (Figure 1C). Immunohistochemical staining with an antibody specific for TORC1 (Figure S3) revealed that TORC1 was almost exclusively located in the cytoplasm of hippocampal neurons (Figure 1D).

### Neuronal activity-dependent nuclear translocation of TORC1

**Figure 2:**
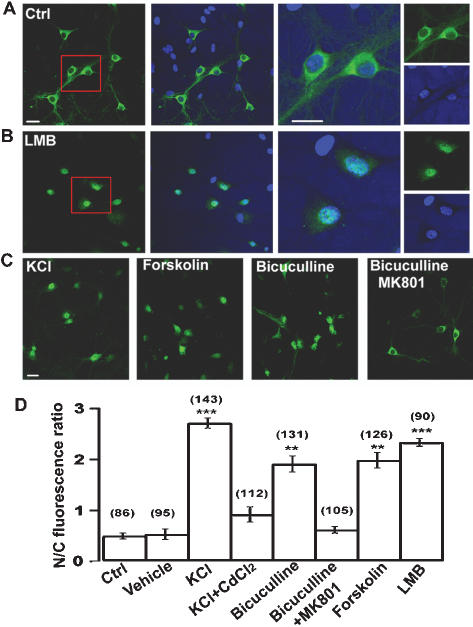
Activity-dependent TORC1 nuclear translocation in cultured hippocampal neurons. (A) Representative immunocytochemistry staining of TORC1 under control condition with lower magnification (left panels). Hochest was used for nuclear staining. Scale bar: 20 µm. High magnification of TORC1 staining and Hochest for nuclear staining (right panels). Scale bar: 20 µm. (B) Representative immunocytochemistry staining of TORC1 after LMB treatment at lower magnification (left panels) and high magnification (right panels). (C) Representative immunocytochemistry staining of TORC1 after treated with KCl, forskolin, bicuculline and bicuculline plus MK801. (D) Statistical analysis of TORC1 distribution after the indicated treatments. Error bars indicate SEM, data in each group were obtained from four independent experiments. The number associated with each column refers to the number of neuron analysed in each treatment. **, *p* < 0.01; ***, *p* < 0.001, compared to control group.

To study whether the subcellular distribution of TORC1 could be regulated by neuronal activity, we performed immunostaining of TORC1 in cultured hippocampal neurons. We observed that TORC1 was mainly distributed in the cytoplasm of cultured hippocampal neurons under control condition (Figure 2A). Treatment with Leptomycin B (LMB), an inhibitor of nuclear protein export [25], led to nuclear accumulation of TORC1 (Figure 2B and 2D). This result was further confirmed by examining the subcellular distribution of EGFP-tagged TORC1 in cultured hippocampal neurons (Figure S4). These data suggested TORC1 undergoes active shuttling between the cytoplasm and nucleus in these neurons. We then examined the distribution of TORC1 by modulating neuronal activity. Increasing Ca^2+^ influx by depolarizing the membrane with high KCl significantly increased the nuclear staining of TORC1, which was blocked by voltage-gated calcium channel blocker CdCl_2_ (Figure 2C and 2D). Elevating excitatory synaptic transmission with GABA_A_ receptor antagonist bicuculline also increased the nuclear accumulation of TORC1, which was prevented by NMDA receptor antagonist MK801 (Figure 2C and 2D). These results indicated that Ca^2+^ influx mediated by voltage-dependent calcium channels and synaptic NMDA receptors can induce TORC1 nuclear accumulation in hippocampal neurons. Increasing intracellular cAMP concentration with forskolin also increased TORC1 nuclear accumulation (Figure 2C and 2D). Taken together, these results suggest that TORC1 undergoes neuronal activity-dependent translocation from the cytoplasm to the nucleus in hippocampal neurons.

### Requirement of TORC1 for CRE-driven gene transcription in neurons

**Figure 3:**
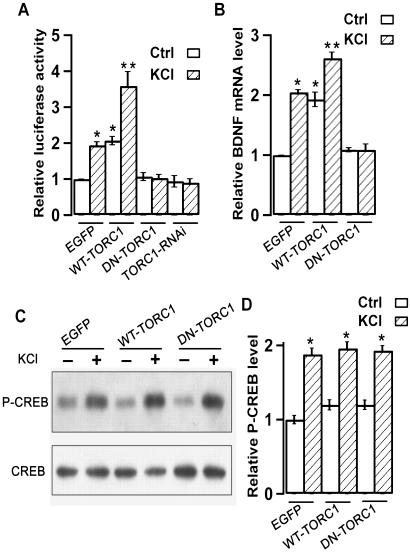
Requirement of TORC1 for CRE-driven gene transcription in cultured hippocampal neurons. (A) Statistical analysis of CRE-luciferase activity from neurons transfected with indicated plasmids that were left unstimulated or stimulated with high KCl. (B) Statistical analysis of BDNF mRNA level from EGFP, WT-TORC1, DN-TORC1 infected hippocampal neurons that were left unstimulated or stimulated with high KCl, GAPDH mRNA of each sample was used as loading control. (C) Representative Western blotting analysis of phospho-CREB (P-CREB) level from EGFP, WT-TORC1, DN-TORC1 infected neurons that were left unstimulated or stimulated with high KCl for 5 min. Protein loading was confirmed by probing the same blot with CREB antibody. (D) Statistical analysis of phospho-CREB (P-CREB) level from EGFP, WT-TORC1, DN-TORC1 infected neurons from four independent experiments. *, *p* < 0.05, compared to non-stimulated control group. In both (A) and (B), data were obtained from three independent experiments, each of which was conducted in triplicate. Error bars indicate SEM. *, *p* < 0.05; **, *p* < 0.01, compared to non-stimulated EGFP group.

In mammalian cell lines TORCs are essential for CREB-dependent gene transcription [20]. Activity-dependent nuclear translocation of TORC1 may be responsible for CREB-mediated gene transcription in neurons. To manipulate the functional interaction of TORC1 with CREB, we generated a dominant-negative form of TORC1 (DN-TORC1) by fusing the N-terminal 44 amino acid of TORC1 with EGFP (Figure S5A), because previous study demonstrated that the N-terminal of TORC1 is essential for binding with bZIP domain of CREB [20], [21], [26]. We further generated a TORC1 knock-down construct (TORC1-RNAi), which efficiently knocked down of TORC1 protein as revealed by cotransfection in cell line (Figure S5B) and endogenous TORC1 in cultured hippocampal neurons (Figure S6). Using CRE-dependent luciferase assay, we examined the role of TORC1 in CREB-driven gene transcription. Transfection of DN-TORC1 blocked KCl-induced elevation of CRE reporter gene transcription (Figure 3A). Knockdown of TORC1 also blocked KCl-induced reporter gene expression (Figure 3A), while overexpressing the wild-type form of TORC1 (WT-TORC1) increased both basal and KCl-induced CRE reporter gene expression (Figure 3A).

To explore the role of TORC1 on endogenous CRE-driven gene transcription in neurons, we performed semi-quantitative RT-PCR analysis for the mRNA level of endogenous BDNF, a well established CREB-targeted gene in neurons [12], [18], [27]. Neurons infected with SFV-EGFP control plasmid showed significant increase of BDNF mRNA level after KCl stimulation (Figure 3B). Overexpressing DN-TORC1 blocked KCl-induced BDNF gene transcription (Figure 3B). In contrast, neurons infected with WT-TORC1 showed significant increase of both basal and KCl-induced BNDF mRNA level (Figure 3B). All modulations did not affect the phosphorylation of CREB at Ser133 after KCl stimulation (Figure 3C and 3D). These results indicated that TORC1 effectively couples neuronal activity to CREB-dependent gene expression in hippocampal neurons.

### TORC1 nuclear translocation correlates with L-LTP induction

**Figure 4:**
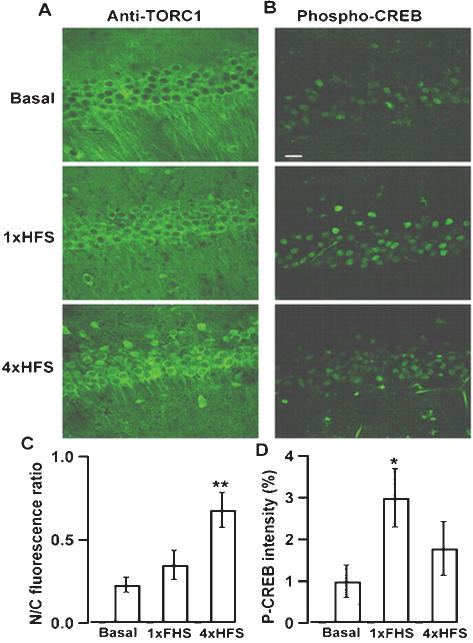
TORC1 nuclear translocation correlates with L-LTP induction. (A). Immunohistochemical staining of TORC1 after basal stimulation (upper panel), 1×HFS (middle panel) and 4×HFS (lower panel). (B) Immunohistochemical staining of phospho-CREB (P-CREB) after basal stimulation (upper panel), 1×HFS (middle panel) and 4×HFS (lower panel). Scale bar, 20 µm. (C). Quantitative analysis of TORC1 immunohistochemical staining after indicated stimulation. (D) Quantitative analysis of phospho-CREB level after indicated stimulation. In both (C) and (D), data were obtained from at least eight slices collected from three independent experiments. *, *p* < 0.05; **, *p* < 0.01 relative to basal group.

Previously studies indicated that the expression of L-LTP correlates with CRE-driven gene transcription, but not CREB phosphorylation [4], [9], suggesting additional CREB coactivator is required for CRE-target gene expression. We investigated the subcellular localization of TORC1 in CA1 neurons of acute hippocampal slices after induction of E-LTP or L-LTP by stimulating the Schaffer collateral pathway. In unstimulated slices, TORC1 was distributed in the cytoplasm as revealed by co-staining with nuclear marker Hochest (Figure 1D). After basal stimulation at 0.033 Hz for 30 min, the TORC1 distribution remained largely cytoplasmic (Figure 4A). One train of high-frequency stimuli at 100 Hz for 1 s (1×HFS) that typically induces E-LTP did not significantly increase the nuclear staining of TORC1 (Figure 4A and 4C). However, marked nuclear accumulation of TORC1 was observed in slices after stimulation with four trains of HFS (4×HFS), a typical L-LTP induction protocol (Figure 4A and 4C). Studies of the phosphorylation level of CREB after the same stimulation protocols showed that significant increase of phospho-CREB level was observed in slices treated with 1×HFS (Figure 4B and 4D) but not with 4×HFS (Figure 4B and 4D), as compared with the basal condition (Figure 4B and 4D). These results indicated that nuclear accumulation of TORC1, but not CREB phosphorylation, correlated with L-LTP induction in hippocampal slices.

### Requirement of TORC1 for L-LTP maintenance

**Figure 5:**
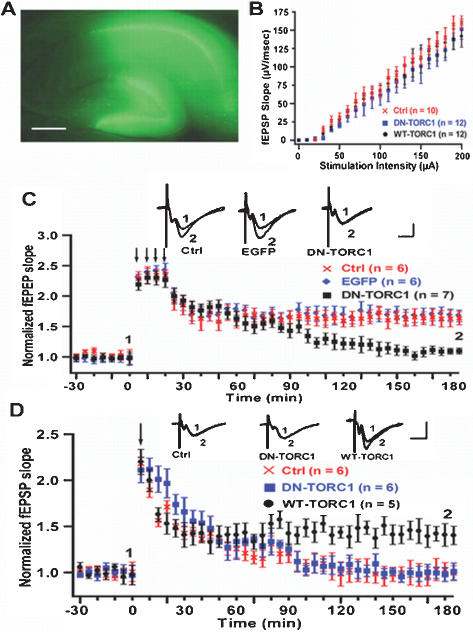
Requirement of TORC1 for L-LTP in hippocampal slices. (A) Representative picture of EGFP infected slice used in electrophysiological recordings. Scale bar: 100 µm. (B) Statistical analysis of input-output ratio from control slices and slices infected with DN-TORC1 or WT-TORC1. (C) DN-TORC1 but not EGFP infection blocked the maintenance of L-LTP as compared with EGFP infected slices and non-infected control slices (percent baseline, at 3 h, DN-TORC1  =  109.2% ± 6.5 vs EGFP  =  178.3 ± 10.2 and Control  =  176.9 % ± 13.4). (D) WT-TORC1 infection switched E-LTP into L-LTP, DN-TORC1 infection did not affect E-LTP (at 3 h, WT-TORC1  =  145.6 % ± 10.7 vs Control  =  102.8 % ± 6.2 and DN-TORC1  =  103.0 ± 7.5). Scale bar: 300 µV, 10 ms. In both (C) and (D), representative fEPSP traces taken at the times indicated (1 and 2) are shown at top, arrows indicate the time of HFS.

To investigate the role of TORC1 in synaptic plasticity of hippocampus, we sterotaxically injected viruses expressing EGFP, WT-TORC1, or DN-TORC1 into the CA1 region of the rat hippocampi. Acute hippocampal slices were prepared 1 d post-infection and electrophysiological recordings were performed in a double-blinded manner. To minimize the contribution of non-specific presynaptic effects, we used only slices in which CA1 but not CA3 neurons were highly infected as shown in Figure 5A. The input-output relationship between uninfected and infected slices did not differ (Figure 5B). Presynaptic paired pulse ratio did no differ between uninfected and infected slices either (Figure S7). These results suggested that viral-mediated infection does not significantly change basal synaptic properties. This result is consistent with previous electrophysiological studies using SFV mediated gene delivery strategy [28]–[31]. We did not observe significant difference between control and DN-TORC1 infected slices in E-LTP induced by 1×HFS (Figure 5D). However, L-LTP induced by 4×HFS was completely abolished in DN-TORC1 infected slices (Figure 5C), while EGPF infected slices show normal L-LTP as compared with non-infected control slices. Interestingly, 1×HFS, which typically induces E-LTP in control slices, evoked LTP with an enhanced and sustained long-lasting phase in slices infected with WT-TORC1 (Figure 5D). These results suggest that TORC1 is required for the maintenance of L-LTP. Furthermore, overexpression WT-TORC1 most likely lowered the threshold for L-LTP induction.

## Discussion

The principal findings of this study are fivefold: 1) the CREB coactivator TORC1 was highly expressed in adult hippocampal neurons; 2) TORC1 underwent neuronal activity-dependent nuclear translocation in responding to elevated intracellular Ca^2+^ and cAMP; 3) TORC1 was required for activity-dependent neuronal CRE-target gene transcription; 4) TORC1 nuclear translocation, but not CREB phosphorylation, correlated with L-LTP induction; 5) TORC1 is required for L-LTP maintenance in rat hippocampus.

TORCs were recently identified as CREB coactivators from human cell lines [20], [22]. Different isoforms of TORCs, TORC1, TORC2 and TORC3 have differential expression pattern in human tissue by cDNA microarray study [21], in contrast to the ubiquitous expression pattern of CREB [17], suggesting that TORCs regulate the function of CREB in stimulus/tissue-specific manner. Indeed, recent study of TORC2 revealed its critical role in regulating glucose homeostasis in liver tissue [24]. In the present study, we examined the expression pattern of TORCs in the central nervous system. We found that TORC1 is highly expressed in the central nervous system; TORC2 was enriched in the cerebellum, while TORC3 was not detectable in the brain. We showed TORC1 was required for CRE-driven gene transcription in the hippcampal neurons.

In the central nervous system, activity-dependent expression of CRE-driven genes is important for neuronal development, synaptic plasticity, and memory formation [4], [8], [12], [32], as well as for pathological states, such as drug addiction [33]. The transcriptional factor CREB has been proposed as a molecular switch in hippocampal synaptic plasticity [8], [11]. The expression of L-LTP correlates with increased CRE-dependent gene expression, as monitored via the activity of a CRE-driven *lacZ* reporter construct in transgenic mice [4]. LTP and long-term memory was defective in mice homozygous for a genetic deletion of αδ isoforms of CREB [8]. Furthermore, expressing a constitutively active form of VP16-CREB facilitated the induction of L-LTP via activation of the transcription of CRE-driven genes [9], [34]. However, other studies failed to demonstrate impairments of L-LTP in CREB mutant mice [35], [36]. In addition, transgenic mice overexpressing a dominant-negative mutant of CREB in amygdala did not show any deficit in LTP or memory [37]. It could be that the role of CREB in LTP may be sensitive to gene dosage and genetic background, and other genes can compensate for loss of CREB in CREB mutant mice [38]. In fact, the CREB partial knockout mice show strong upregulation of other CRE binding transcription factors [39], [40]. Our findings that TORC1 is required for L-LTP in acute hippocampal slices suggest that CREB-mediated gene expression is critical for L-LTP because CREB is the only identified partner for TORCs. It is also possible that TORC1 works through some other transcriptional factors besides CREB for CRE-target gene expression and L-LTP. For this issue, it would be interesting to examine the role of TORC1 in L-LTP in mice without functional CREB in future experiments.

The molecular mechanisms whereby synaptic signals propagate to the nucleus to maintain genes expression for synaptic potentiation were unclear. Previous studies indicates that nuclear translocation of phospho-MAP kinase and calmodulin contributes to CREB phosphorylation in cultured hippocampal neuron and slices [41]–[45]. Phosphorylation of CREB and the recruitment its coactivators CBP and p300 to the CRE element, promote the transcription of downstream genes [14]–[18]. The convergence of cAMP and Ca^2+^ signals at the level of CREB Ser133 phosphorylation provides a plausible mechanism for cooperativity among diverse signals for CRE-target gene expression and L-LTP. However several groups reported that some extracellular stimuli capable of phosphorylating CREB at Ser-133, fail to induce CREB-dependent gene expression [12], [46]. Previous study [4] and our data revealed discrepancy in CREB phosphorylation between 1×HFS and 4×HFS in hippocampal slices. It is possible that 4×HFS activated protein phosphatase that dephosphorylates CREB due to prolonged strong synaptic activity-induced calcium influx, while only very little phosphatase was activated after 1×HFS. In fact, previous work indicated that the duration of CREB phosphorylation could be regulated by protein phosphatases, depending on the stimulation pattern [47], [48] and the sites of calcium entry. For example, calcium influx via NMDA receptor could dephosphorylate KCl-induced CREB phosphorylation [49]. Synaptic and extrasynaptic NMDA receptors have opposite roles on CREB phosphorylation [50]. Our findings suggest that nuclear translocation of the CREB coactivator TORC1 is required for neuronal activity-dependent CRE-target gene expression. Moreover, TORC1 nuclear accumulation, but not CREB phosphorylation, correlated with L-LTP induction. Taken together, these findings suggest that CREB has to recruit TORC1 to initiate CRE-target gene expression beside Ser133 phosphorylation.

In summary, our results suggest that TORC1 acts as the coincidence detector for sensing intracellular Ca^2+^ and cAMP changes in neurons and is translocated to the nucleus to support persistent CREB-target gene transcription. These results may provide an alternative mechanism to explain the discrepancy between transient CREB phosphorylation and persistent CRE-driven gene transcription. We demonstrated that TORC1 is a critical component for activity-dependent CREB target gene expression and the maintenance of L-LTP in the hippocampus.

## Materials and Methods

### TORC1 gene cloning and constructs preparation

To clone Rat TORC1 coding sequence, a pair of degenerative primers is designed according to mouse TORC1 (Genbank accession: BC080308): Forward: 5′- ATGGCGACTTCGAACAATCCGCGGA -3′; Reverse, 5′- TCACAGGCGGTCCATTCGGAAGGT -3′. Using above primers, a cDNA sequence was amplified by RT-PCR using hippocampal total RNA from adult male Sprague-Dawley (SD) rat brain, and inserted into pGEM-T easy vector to generate pGEM-rTORC1. A verified sequence has been submitted into NCBI database (GenBank accession: 108865216). To clone N-terminal tagged Flag-TORC1, a fragment was amplified using pGEM-rTORC1 as template and inserted into pcDNA3 between HindIII- and EcoRI, and it was named pcDNA3-flag-rTORC1. To clone TORC1-GFP fusion protein expression construct, TORC1 coding sequence was amplified from pGEM-rTORC1 and inserted into pEGFP-N1 vector (Clontech) between HindIII and EcoRI to generate prTORC1-EGFP. For dominant negative TORC1 expression, a N-terminal 44 amino acid coding sequence was inserted into pEGFP-N1 between EcoRI and HindIII as described [22] to generate pN44-rT1-EGFP. The primers for pcDNA3-flag-rTORC1 are: Forward, 5′-CCCAAGCTTATGGATTACAAGGATGACGACGATAAGGCGACTTCGAACAATCCGCGGA-3′; Reverse, 5′-CCGGAATTCTCACAGGCGGTCCATTCGGAA-3′. Primers for prTORC1-EGFP are: Forward, 5′-GCCCAAGCTTATGGCGACTTCGAACAATCCGCG-3′; Reverse, 5′-CCGGAATTCGCAGGCGGTCCATTCGGAAGGT-3′.

### TORC1 RNAi preparation

To screen RNAi targeting sequence, three hairpin DNA oligos targeting rat TORC1 (Shanghai Genechem Co., Ltd. China) as well as a control hairpin DNA targeting luciferase were cloned into pLVTHM vector (kindly gift from Dr. Trono). For knockdown efficiency test, pLVTHM-shRNA was co-transfected with targeting DNA pcDNA3-flag-rTORC1 (ratio, 3∶1) into BHK-21 cell. Forty eight hours later, cells were harvested and lysed for Western blotting analysis. One of the most efficient shRNA sequence (22-mer sequence, 5′-CCGACATCATGGGCTTGTGGAC-3′) was used in functional assays in this paper.

### Virus vectors and packaging of virions

The Semliki Forest Virus (SFV)-based vector and helper vector were kindly provided by Dr. Kenneth Lundstrom (Hoffmann La Roche, Basel). To construct pSFV(pd)-EGFP, a DNA fragment encoding enhanced green fluorescent protein (EGFP; Clontech,) was amplified by PCR and digested to generate a 5′ Xho I and 3′ Spe I sites, and inserted into pSFV(pd) vector. The DNA fragment encoding full-length rat torc1 and 44 amino acids in N-termini of rat torc1 were amplified by PCR to generate 5′ SpeI and 3′ Not I sites, and then inserted into pSFV(pd)-EGFP vector to produce pSFV(pd)–TORC1-EGFP and pSFV(pd)-DN-T1-EGFP. Virus packaging was performed as previously described [51].

### Primary neuronal culture, transfection, and stimulation

Hippocampal neuron cultures were prepared from E18 SD rat embryos and maintained for 15–21 d *in vitro* (DIV) as previously described [29]. For BDNF mRNA analysis, neurons at DIV 5–6 were infected with activated virions as indicated. After 16–18 h infection, neurons were stimulated with 50 mM KCl for 4 h, then collected for RT-PCR. For immunocytochemical study, neurons at DIV 14–15 were stimulated either with agonists or in combination with antagonists as follows: KCl (70 mM), CdCl_2_ (50 µM) for 5 min, forskolin (25 µM) for 15 min, bicuculline (50 µM), MK-801 (10 µM ) for 30 min, LMB (10 ng/ml ) for 1 h.

### Semi-quantitative RT-PCR analysis

For culture assay, total RNA of SFV-infected DIV 6–7 hippocampal neurons were extracted with TRIzol reagent (Invitrogen). 1.5 µg total RNA was reverse transcribed, and 1/20 of the RT products were used for PCR amplification. For TORC1 tissue expression pattern assay, total RNA from the cerebral cortex, hippocampus, and cerebellum of SD rat were extracted and used for RT-PCR. Primers used are: BDNF, Forward, 5-ACGGTCACAGTCCTGGAGAAA -3′, Reverse, 5′- TTGGGTAGTTCGGCATTGCGA –3; TORC1, Forward: 5′-GCACAACCAGAAGCAGGCG-3′, Reverse: 5′-CAGGACTTGGGCCTGGAAC-3′; GAPDH, Forward, 5′- ATGCCCCCATGTTTGTGATGG -3′, Reverse, 5′- TGGTCATGAGCCCTTCCACGA -3. BDNF mRNA level in each lane was normalized with GAPDH mRNA and quantified with ImageQuant 5.2 (GE Healthcare).

### *In Situ* Hybridization

*In situ* hybridization was performed as previously described [52]. Briefly, SD rats were deeply anaesthetized and perfused with 4% paraformaldehyde in 0.1 M phosphate buffer saline (DEPC-PBS; pH 7.4). The whole brains were removed and placed into 4% paraformaldehyde in 0.1 M phosphate buffer (DEPC-PBS; pH 7.4) for 4 hours. Brains were dehydrated in gradient of sucrose (15%, 30%, dissolved with DEPC-PBS). Brains were cut into 20 µm thin coronary slices in −20°C with Cryostat CM1900 (Leica, Germany) and mounted onto no-RNAase silane-coated slides (Fisher Scientific, USA), stored at −20°C.The PCR products of rat TORC1 (forward: 5′-GCACAACCAGAAGCAGGC-3, Reverse:5′-CAGGACTTGGGCCTGGAAC-3′) were ligated with pGEM-T Easy vector. The plasmid were linearized for in vitro RNA transcription to prepare antisense and senese DIG-RNA probes using a SP6/T7 transcription kit (Roche). Hybridization was performed following the standard protocol, anti-DIG-AP-Fab fragments, NBT/BCIP (Boehringer Mannheim) were used for detection.

### CRE-dependent luciferase reporter assay

For all reporter experiments, firefly luciferase plasmid pTAL-Cre (Clonetech) were cotransfected with the RL-SV40 vector (Promega), which expresses Renilla luciferase as an internal control, using calcium phosphate transfection into cultured hippocampal neuron at DIV 4–5 as previously described [53]. Forty hours following transfection, neurons were stimulated with KCl (50 mM) for 8 h. The luciferase assays were performed using a Dual Luciferase Assay kit (Promega) according to the manufacturer's instructions.

### Antibodies preparation and reagents

All reagents were purchased from Sigma unless otherwise indicated. TORC1- and TORC3-antisera were raised against human TORC1 peptide (551–650) and TORC3 peptide (480–619), respectively. Because these antisera have cross-reaction with both TORC1 and TORC3, the IgG was purified from the anti-TORC1 serum using the TORC3 peptide. The resultant anti-TORC1/3 IgG could recognize both TORC1 and TORC3 with equal efficiency. The above peptides were prepared as GST-fusion peptides in E. coli using a pGEX-6P3 expression vector and cleaved from GST by PreScission Protease (Amersham Biosciences).

### Northern Blotting

Total cellular RNA was extracted from hippocampus, cerebral cortex and cerebellum of adult rats by using the TRIzol reagent (Invitrogen). RNA (20 µg) from each sample was loaded onto a 1.5% agarose/formaldehyde gel, transferred to a nylon membrane (Amersham) after electrophoresis, and hybridized with 32P-labeled cDNA probes spanning 48 nt to 647 nt in the coding sequence of TORC1. Equivalent loading of RNA in all lanes were confirmed by 28S and 18S RNA.

### Western Blotting

Brain tissues or cultured neurons were lysed and protein concentrations were determined by the Lowry method. Approximately 10∼20 µg of each sample was loaded onto each lane and size-fractionated on 9% SDS-PAGE. After electrotransfer, membrane was blocked with 5% milk in 0.05% Tween-20 for 1 h. The blots were probed with antibodies diluted as follows: rabbit anti-TORC1/3 (1∶4000), rabbit anti-phospho-CREB (1∶1000; Cell Signaling Technology), rabbit anti-CREB (1∶1000; Cell Signaling Technology), and mouse anti-Actin (1∶5000; Santa Cruze Biotechnology) antibodies. Goat anti-rabbit or mouse IgG coupled to HRP (1∶5000; Amersham) were used as the secondary antibodies. Bands were visualized by ECL plus Western blotting detection system (Amersham). Prestained proteins (17 to 108kDa; RYM-TECH) were used as marker. Quantitative analysis of phosphor-CREB level was performed by normalizing to total CREB level using ImageQuant 5.2 (GE Healthcare).

### Immunostaining

Slice Immunohistochemical experiments were performed as previously described [43]. Hippocampal slices were prepared and treated with tetanic stimulation as indicated in the electrophysiological experiments. Thirty min after each treatment, slices were fixed in ice-cold 4% paraformaldehyde for 60 min, permeailized in 0.3% Triton X-100 for 60 min, blocked with 10% goat serum for 60 min. Slices were then incubated with rabbit anti-phospho-CREB (1∶200 Cell Signaling Technology), or rabbit anti-TORC1/3 IgG (1∶500) in 10% goat serum in PBS at 4°C for 36 h. After thoroughly wash with PBS, slices were probed with Alex 488 conjuated goat anti-rabbit secondary antibody at 4°C for 36 h (1∶2000, Molecular probes). The slices were viewed using a Zeiss two photon laser confocal-scanning system (Zeiss LSM 510). Images were taken using a 40×water immersion objective with a numerical aperture of 0.8. Each image was collected by averaging four scans. For Immunocytochemical staining in cultured hippocampal neurons, neurons were fixed in ice-cold 4% paraformaldehyde, permeabilized in 0.1% Triton X-100 and blocked in 5% goat serum in PBS. The primary anti-TORC1/3 antibody (1∶4000) and Alex 488 conjuated goat anti-rabbit secondary antibody (1∶5000) were used for experiments, Hochest 33342 (Sigma) was used to validate the morphological identification of nuclei. Image were taken using a 40×oil immersion objective with a numerical aperture of 1.3. Immunofluorescence intensity ratio of nuclear to cytosol (N/C fluorescence ratio) both in hippocampal slices and cultured hippocampal neurons were analysed as previously described [54], the fluorescence intensity per unit area in the nucleus versus cytosol was counted by the Image Pro Plus software (Media Cybernetics).

### Animal stereotaxic surgery

The use and care of animals in this study is approved by the Institutional Animal Care and Use Committee of the Institute of Neuroscience. Postnatal day 21–28 SD rats (45–65 g) were fully anesthetized with 5% Choral Hydrate and prepared for stereotaxic injection as previously described [28]. Activated virons (1 µl) were infused into the hippocampus of rats unilaterally (−3 mm posterior to bregma, −1.8 mm lateral to the midline) using a stainless steel cannula (Plastic One) at a depth of 2.9 mm and controlled with a Harvard Apparatus pump at a flow rate of 0.1 µl/min.

### Slice preparation and electrophysiology recording

Hippocampal slices were prepared 18∼24 h after infection as described [28]. Briefly, rats were deeply anaesthetized, brains were rapidly removed and transverse hippocampal slices (400 µm thickness) were cut using a vibrating blade microtome (Leica VT 1000S) in ice-cold artificial cerebrospinal fluid (ACSF containing 119 mM NaCl, 2.3 mM KCl, 1.3 mM MgSO_4_, 2.5 mM CaCl_2_, 26.2 mM NaHCO_3_, 1 mM NaH_2_PO_4_, and 11 mM glucose) that was bubbled continuously with carbogen (95%O_2_/5%CO_2_). After equilibration for 60 min at 34°C, slices were transferred to a submerged chamber perfused continuously with arbogenated ACSF containing bicuculline (10 µM) at 30°C via an automatic temperature cotronller (Warner Instrument Corporation). Recording of fEPSP in the CA1 region of infected hippocampal slices were performed as previously described [9], [42]. Both the stimulating and recording electrodes (filled with ACSF) were placed in the stratum radiatum of CA1 area. Excitatory postsynaptic responses were evoked by stimulating the Schaffer collateral commissural pathway via a constant current pulse (0.05 ms) delivered by Master-8 stimulator (AMPI) through a stainless bipolar electrode (100 µm diameter). The stimulation intensity (0.05 ms duration) was adjusted to give fEPSP slopes approximately 40% of the maximum, and baseline responses were elicited once per minute at this intensity. L-LTP was produced with four 100 Hz one sec tetani presented at 5 min intervals. E-LTP was produced with one 100 Hz one sec tetanus. Slopes of fEPSP were plotted every 5 min by averaging five consecutive slopes. Only one slice from each rat was used for recording, “n” indicates the number of slices. Data analyses were performed using Clampfit 8.0 and IGOR PRO version 4.09 (WaveMetrics).

### Statistical analysis

Statistical data are given as mean ± SEM. The significance of differences was determined using Student's *t* test as compared to control group using Prism 4.0 software.

## Supporting Information

Figure S1 Expression pattern of TORCs in adult rat brain regions. (A). RT-PCR analysis of TORC1, TORC2 and TORC3 mRNAs in adult rat hippocampus, cerebral cortex and cerebellum. (B) Western blotting analysis of TORC2 protein in adult rat hippocampus, cerebral cortex and cerebellum.
(0.11 MB DOC)

Figure S2 Sequence alignment of human (hTORC1), mouse (mTORC1) and rat (rTORC1) TORC1 protein. Characters in yellow indicate the conserved amino acid in all three species, characters in blue indicates the conserved amino acid in two of three species.
(2.22 MB DOC)

Figure S3 Specificity of TORC1 antibody. TORC1 overexpression panel indicated the lysate from BHK-21 cells overexpressed with TORC1 plasmid. A full blot was presented with a protein marker ranging from 17 to 108 kDa.
(0.61 MB DOC)

Figure S4 Representative image of EGFP-tagged TORC1 in cultured hippocampal neurons 16 hrs after infection with EGFP tagged WT-TORC1. (A) Non-treated control neurons. (B) LMB treated neuorns. Scale bar: 20 µm.
(0.70 MB DOC)

Figure S5 Schematic graph of DN-TORC1 construction and TORC1 RNAi efficiency examination. (A) Generation of a DN-TORC1 by fusing the 44 amino acids from N-terminal CREB binding domain of TORC1 with a full length EGFP. (B) Western blotting analysis of lysate from TORC1 overexpressed BHK-21 cells co-transfected with either control scramble shRNA or TORC1 shRNA. Blot was probed with anti-TORC1 antibody, stripped and re-probed with beta-actin antibody as loading control.
(0.18 MB DOC)

Figure S6 TORC1 RNAi efficiency examination in primary cultured hippocampal neuron. (A) Anti-TORC1 staining of hippocampal neurons transfected with control scramble shRNA. (B) Anti-TORC1 staining of hippocampal neurons transfected with TORC1 shRNA. In both (A) and (B), neurons were fixed for staining 72 hrs after transfection, transfected neurons were indicated by EGFP fluorescence. Scale bar: 20 µm.
(2.86 MB DOC)

Figure S7 Comparison of Paired pulse ratio (PPF) from control slices and slices infected with DN-TORC1 or WT-TORC1. Representative superimposed traces of PPF from Ctrl slice (A), WT-TORC1 slice (B) and DN-TORC1 slice (C). (D) Statistical analysis of PPF from these slices. Data were presented as the mean {plus minus} SEM of the facilitation of the second response relative to the first response. Scale bar: 200 µV, 50 ms.
(0.76 MB DOC)
