# Supplementary material for: Requirement of TORC1 for Late-Phase Long-Term Potentiation in the Hippocampus
Source: PLoS One. 2006 Dec 20;1(1):e16. doi: 10.1371/journal.pone.0000016 (PMC1762377; doi:10.1371/journal.pone.0000016)
Supplement: Figure S1 — Expression pattern of TORCs in adult rat brain regions. (A). RT-PCR analysis of TORC1, TORC2 and TORC3 mRNAs in adult rat hippocampus, cerebral cortex and cerebellum. (B) Western blotting analysis of TORC2 protein in adult rat hippocampus, cerebral cortex and cerebellum. (0.11 MB DOC) [file pone.0000016.s001.doc]

**Supporting figure S1**

**
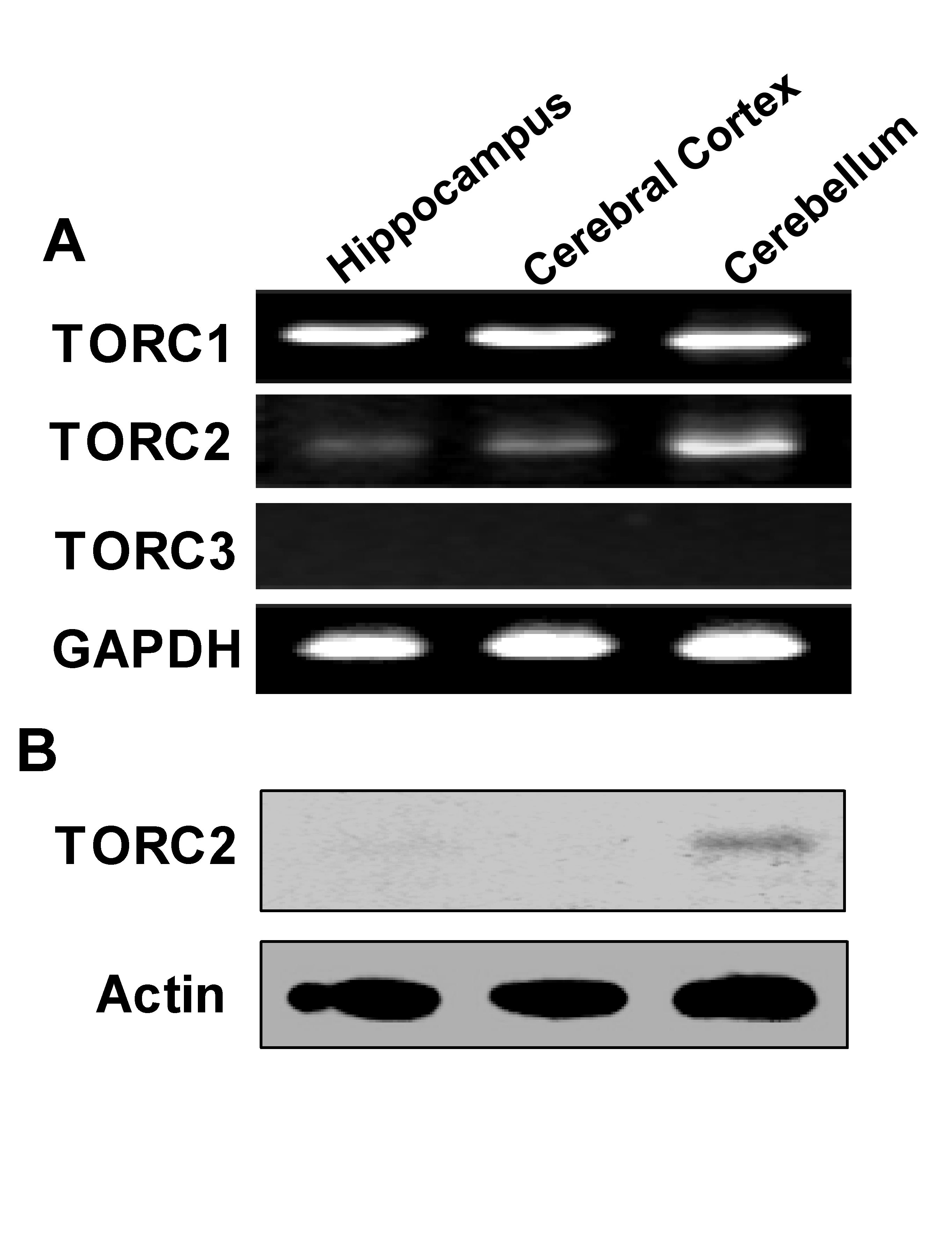
**

**Figure S1.** Expression pattern of TORCs in adult rat brain regions. (A). RT-PCR analysis of TORC1, TORC2 and TORC3 mRNAs in adult rat hippocampus, cerebral cortex and cerebellum. (B) Western blotting analysis of TORC2 protein in adult rat hippocampus, cerebral cortex and cerebellum.
