# Supplementary material for: Requirement of TORC1 for Late-Phase Long-Term Potentiation in the Hippocampus
Source: PLoS One. 2006 Dec 20;1(1):e16. doi: 10.1371/journal.pone.0000016 (PMC1762377; doi:10.1371/journal.pone.0000016)
Supplement: Figure S2 — Sequence alignment of human (hTORC1), mouse (mTORC1) and rat (rTORC1) TORC1 protein. Characters in yellow indicate the conserved amino acid in all three species, characters in blue indicates the conserved amino acid in two of three species. (2.22 MB DOC) [file pone.0000016.s002.doc]

**Supporting figure S2**


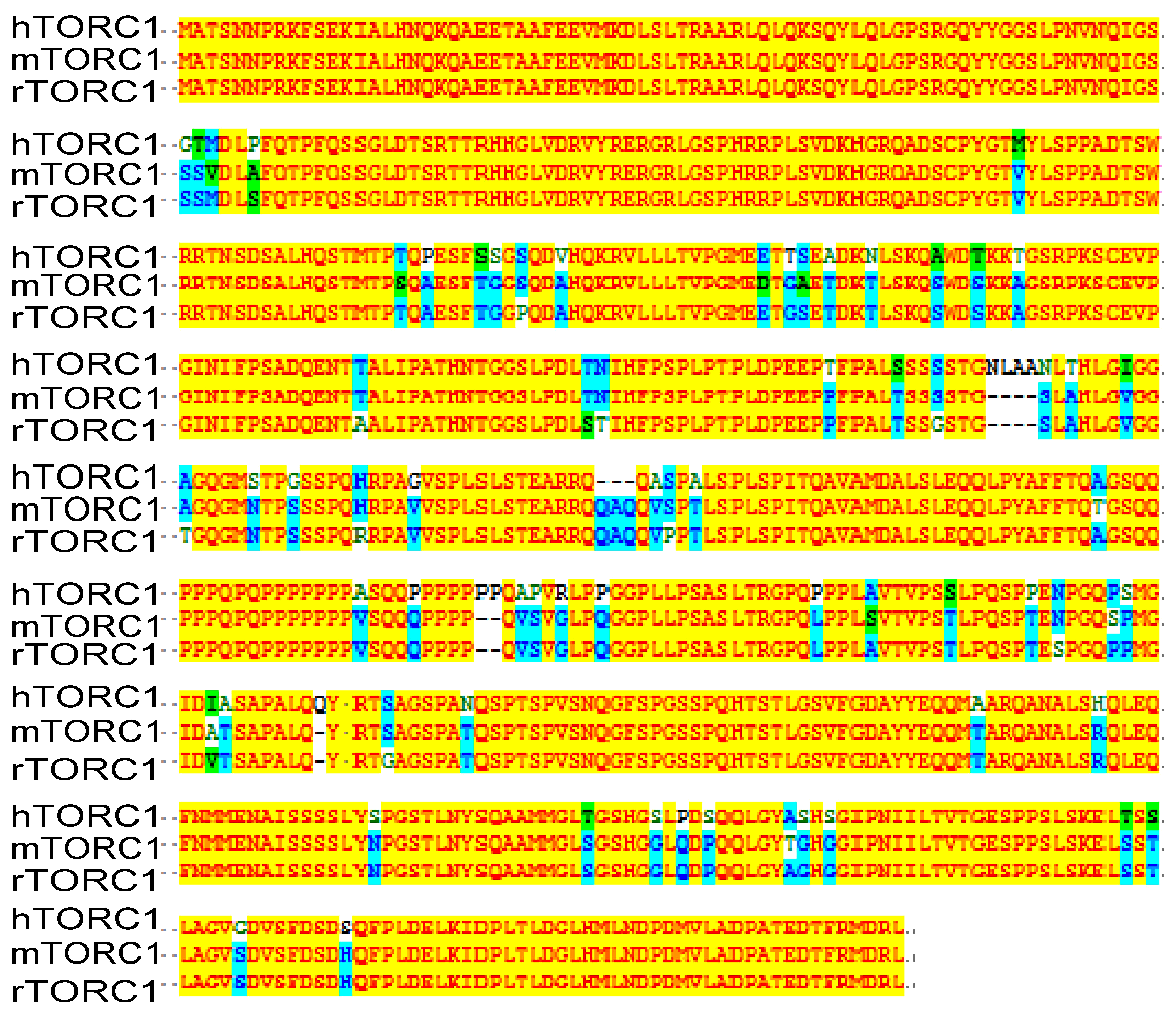


**Figure S2.** Sequence alignment of human (hTORC1), mouse (mTORC1) and rat (rTORC1) TORC1 protein. Characters in yellow indicate the conserved amino acid in all three species, characters in blue indicates the conserved amino acid in two of three species.
