# Supplementary material for: Requirement of TORC1 for Late-Phase Long-Term Potentiation in the Hippocampus
Source: PLoS One. 2006 Dec 20;1(1):e16. doi: 10.1371/journal.pone.0000016 (PMC1762377; doi:10.1371/journal.pone.0000016)
Supplement: Figure S3 — Specificity of TORC1 antibody. TORC1 overexpression panel indicated the lysate from BHK-21 cells overexpressed with TORC1 plasmid. A full blot was presented with a protein marker ranging from 17 to 108 kDa. (0.61 MB DOC) [file pone.0000016.s003.doc]

**Supporting figure S3**

**
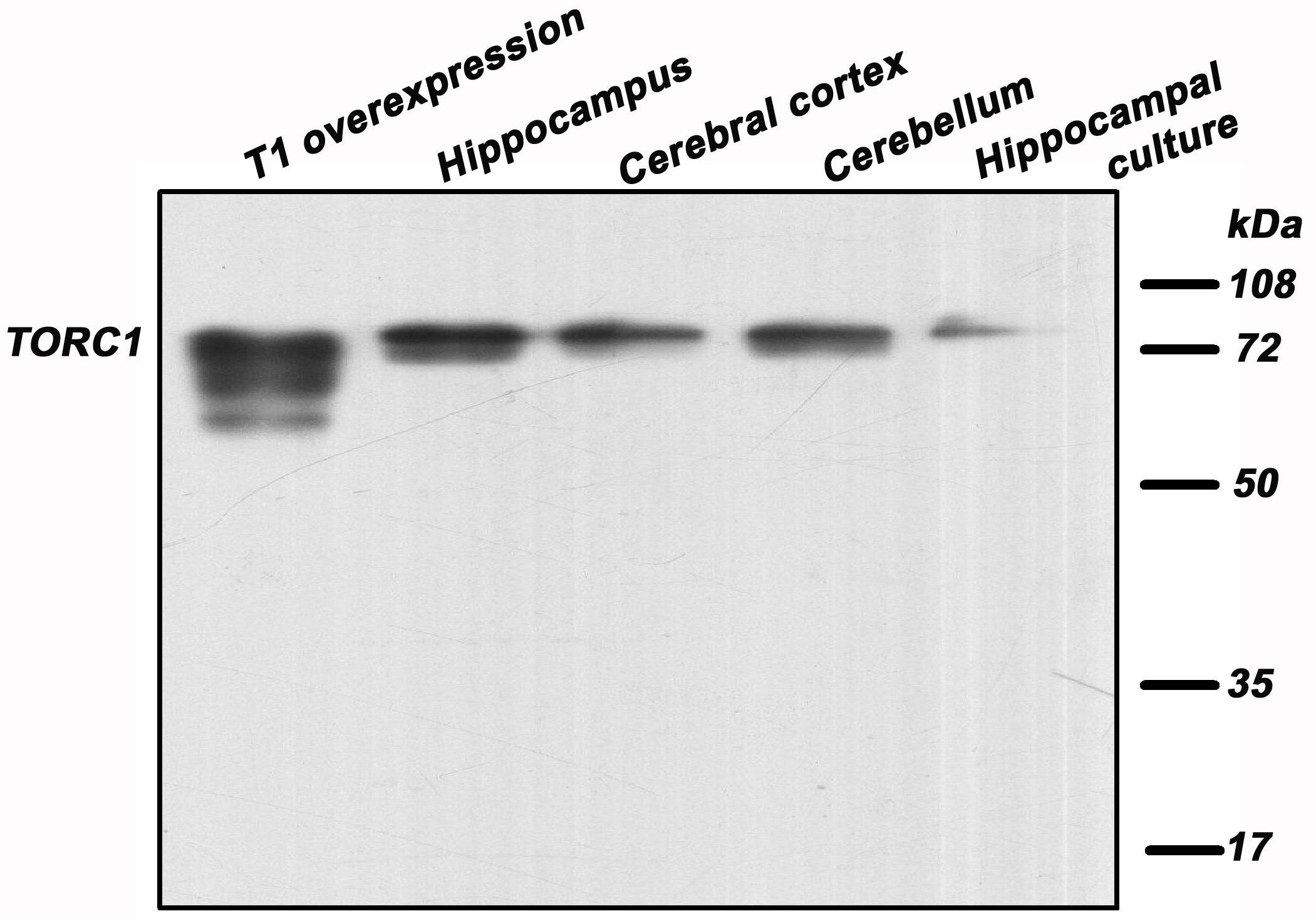
**

**Figure S3.**  Specificity of TORC1 antibody. TORC1 overexpression panel indicated the lysate from BHK-21 cells overexpressed with TORC1 plasmid. A full blot was presented with a protein marker ranging from 17 to 108 kDa.
