# Supplementary material for: Requirement of TORC1 for Late-Phase Long-Term Potentiation in the Hippocampus
Source: PLoS One. 2006 Dec 20;1(1):e16. doi: 10.1371/journal.pone.0000016 (PMC1762377; doi:10.1371/journal.pone.0000016)
Supplement: Figure S4 — Representative image of EGFP-tagged TORC1 in cultured hippocampal neurons 16 hrs after infection with EGFP tagged WT-TORC1. (A) Non-treated control neurons. (B) LMB treated neuorns. Scale bar: 20 µm. (0.70 MB DOC) [file pone.0000016.s004.doc]

**Supporting figure S4**

**
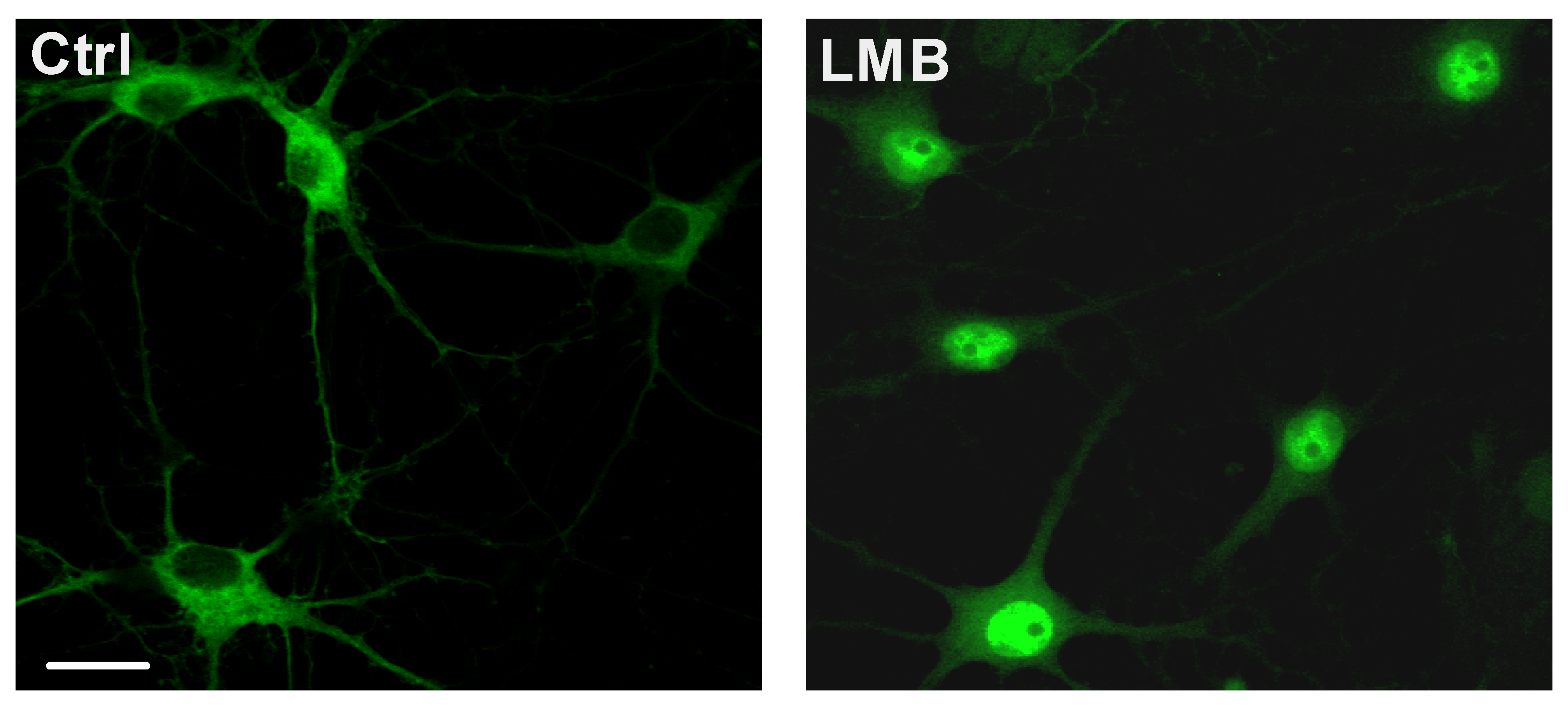
**

**Figure S4.** Representative image of EGFP-tagged TORC1 in cultured hippocampal neurons 16 hrs after infection with EGFP tagged WT-TORC1. (*A*) Non-treated control neurons. (*B*) LMB treated neuorns. Scale bar: 20 μm.
