# Supplementary material for: Requirement of TORC1 for Late-Phase Long-Term Potentiation in the Hippocampus
Source: PLoS One. 2006 Dec 20;1(1):e16. doi: 10.1371/journal.pone.0000016 (PMC1762377; doi:10.1371/journal.pone.0000016)
Supplement: Figure S5 — Schematic graph of DN-TORC1 construction and TORC1 RNAi efficiency examination. (A) Generation of a DN-TORC1 by fusing the 44 amino acids from N-terminal CREB binding domain of TORC1 with a full length EGFP. (B) Western blotting analysis of lysate from TORC1 overexpressed BHK-21 cells co-transfected with either control scramble shRNA or TORC1 shRNA. Blot was probed with anti-TORC1 antibody, stripped and re-probed with beta-actin antibody as loading control. (0.18 MB DOC) [file pone.0000016.s005.doc]

**Supporting figure S5**

**
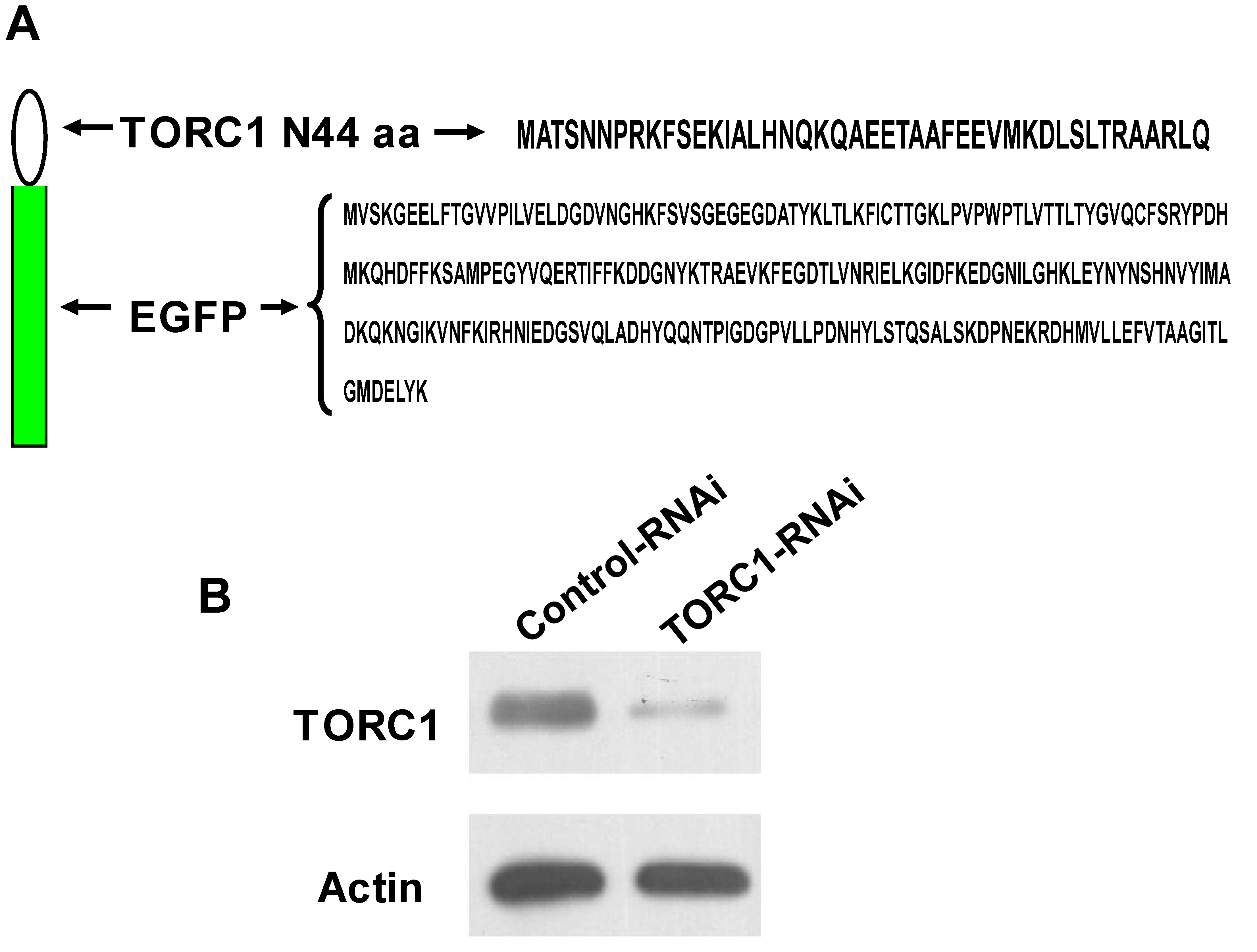
**

**Figure S5.** Schematic graph of DN-TORC1 construction and TORC1 RNAi efficiency examination. (*A*) Generation of a DN-TORC1 by fusing the 44 amino acids from N-terminal CREB binding domain of TORC1 with a full length EGFP. (*B*) Western blotting analysis of lysate from TORC1 overexpressed BHK-21 cells co-transfected with either control scramble shRNA or TORC1 shRNA. Blot was probed with anti-TORC1 antibody, stripped and re-probed with beta-actin antibody as loading control.
