# Supplementary material for: Requirement of TORC1 for Late-Phase Long-Term Potentiation in the Hippocampus
Source: PLoS One. 2006 Dec 20;1(1):e16. doi: 10.1371/journal.pone.0000016 (PMC1762377; doi:10.1371/journal.pone.0000016)
Supplement: Figure S6 — TORC1 RNAi efficiency examination in primary cultured hippocampal neuron. (A) Anti-TORC1 staining of hippocampal neurons transfected with control scramble shRNA. (B) Anti-TORC1 staining of hippocampal neurons transfected with TORC1 shRNA. In both (A) and (B), neurons were fixed for staining 72 hrs after transfection, transfected neurons were indicated by EGFP fluorescence. Scale bar: 20 µm. (2.86 MB DOC) [file pone.0000016.s006.doc]

**Supporting figure S6**

**
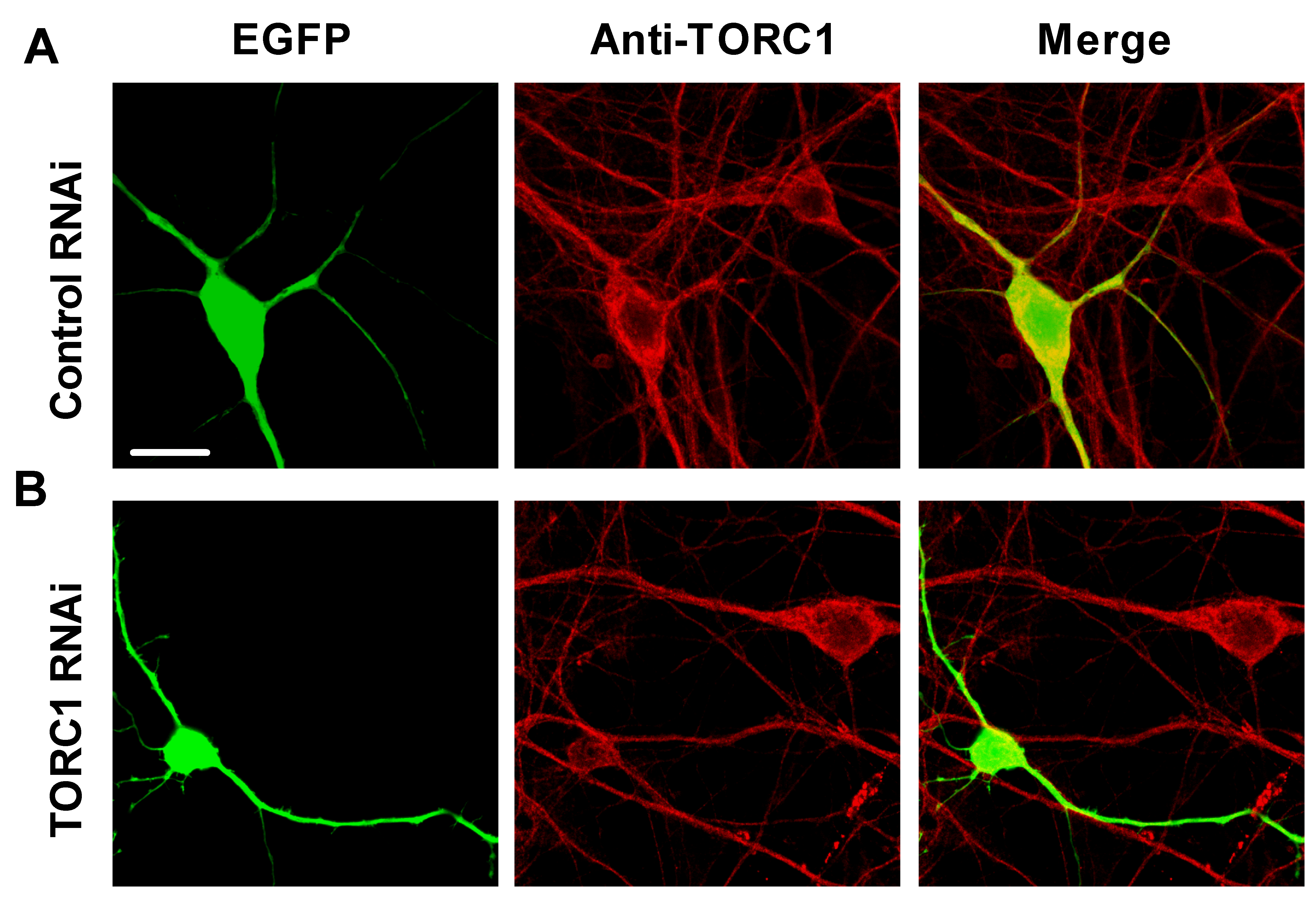
**

**Figure S6.** TORC1 RNAi efficiency examination in primary cultured hippocampal neuron. (*A*) Anti-TORC1 staining of hippocampal neurons transfected with control scramble shRNA. (*B*) Anti-TORC1 staining of hippocampal neurons transfected with TORC1 shRNA. In both (*A*) and (*B*), neurons were fixed for staining 72hrs after transfection, transfected neurons were indicated by EGFP fluorescence. Scale bar: 20 μm.
