# Supplementary material for: Requirement of TORC1 for Late-Phase Long-Term Potentiation in the Hippocampus
Source: PLoS One. 2006 Dec 20;1(1):e16. doi: 10.1371/journal.pone.0000016 (PMC1762377; doi:10.1371/journal.pone.0000016)
Supplement: Figure S7 — Comparison of Paired pulse ratio (PPF) from control slices and slices infected with DN-TORC1 or WT-TORC1. Representative superimposed traces of PPF from Ctrl slice (A), WT-TORC1 slice (B) and DN-TORC1 slice (C). (D) Statistical analysis of PPF from these slices. Data were presented as the mean {plus minus} SEM of the facilitation of the second response relative to the first response. Scale bar: 200 µV, 50 ms. (0.76 MB DOC) [file pone.0000016.s007.doc]

**Supporting figure S7**

**
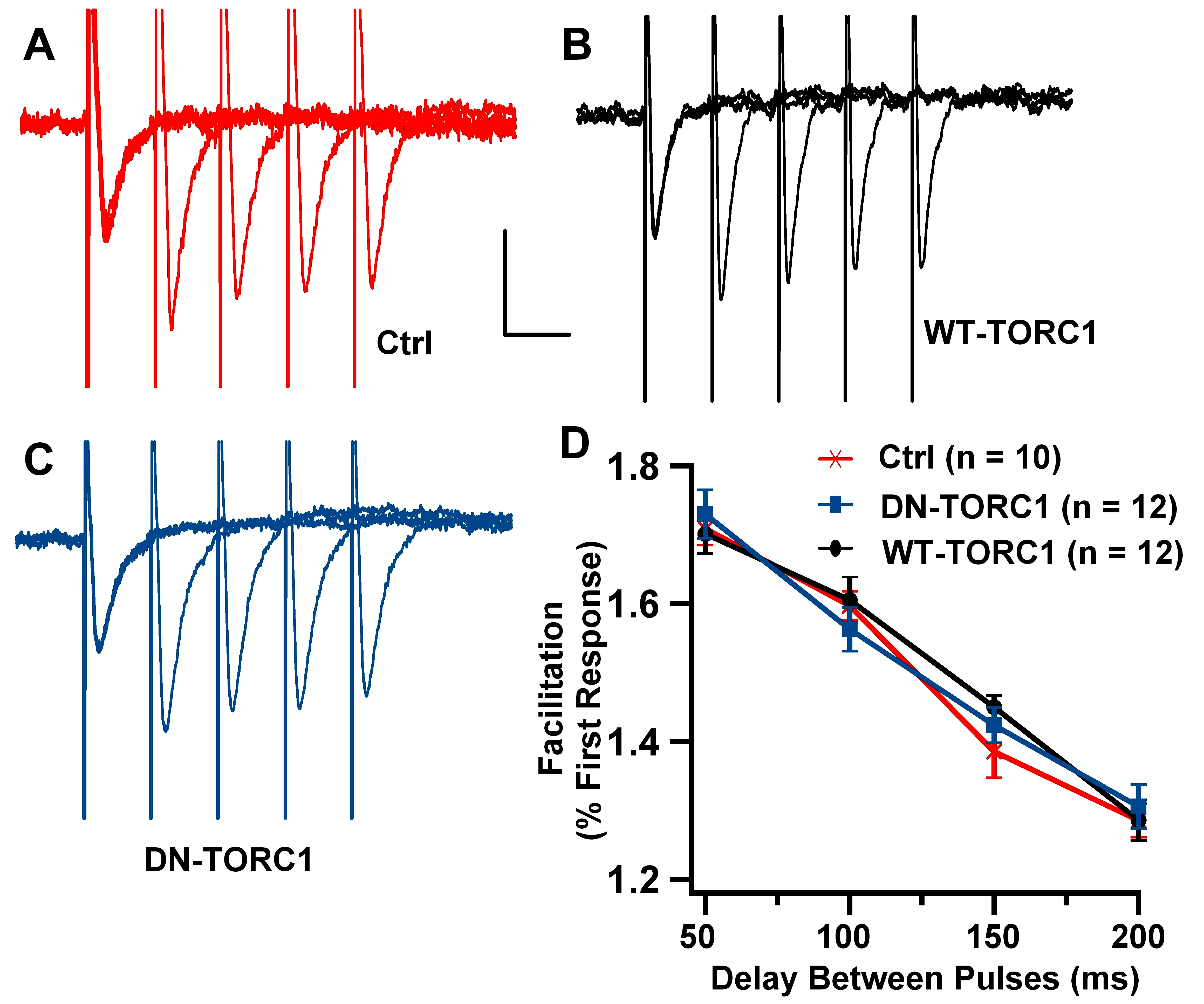
**

**Figure S7.** Comparison of Paired pulse ratio (PPF) from control slices and slices infected with DN-TORC1 or WT-TORC1. Representative superimposed traces of PPF from Ctrl slice (A), WT-TORC1 slice (B) and DN-TORC1 slice (C). (D) Statistical analysis of PPF from these slices. Data were presented as the mean ± SEM of the facilitation of the second response relative to the first response. Scale bar: 200 μV, 50 ms.
